# Dieffenbachia-Induced Transient Crystalline Keratopathy: A Case Report and Review of Previously Reported Cases

**DOI:** 10.7759/cureus.21146

**Published:** 2022-01-12

**Authors:** Su Huan Chong, Tun Wang CH'NG, Mushawiahti Mustapha

**Affiliations:** 1 Ophthalmology, Pusat Perubatan Universiti Kebangsaan Malaysia, Kuala Lumpur, MYS; 2 Ophthalmology, Selayang Hospital, Ministry of Health Malaysia, Batu Caves, MYS; 3 Ophthalmology, Hospital Raja Permaisuri Bainun, Ipoh, MYS; 4 Ophthalmology, Pusat Perubatan Universiti Kebangsaan Malaysia, Kuala lumpur, MYS

**Keywords:** plant extracts, corneal diseases, cornea, calcium oxalate, araceae

## Abstract

We report a case of transient crystalline keratopathy induced by contact with *Dieffenbachia* plant sap and review previous reports on plant-induced crystalline keratopathy. A 27-year-old man with underlying diabetes mellitus presented with ocular pain, redness, and tearing after *Dieffenbachia* plant sap accidentally entered his right eye while he had been cutting the grass one day prior to presentation. The visual acuity of his right eye was 6/9. His conjunctiva was injected with an epithelial defect over the inferior conjunctiva. There were fine needle-like oxalate crystals in the epithelial and stromal layer of the cornea inferiorly. He was treated with topical dexamethasone and levofloxacin. The crystals disappeared from the cornea when he was reviewed one month later. He maintained good visual acuity, and no corneal opacity was noted. In conclusion, patients with crystalline keratopathy after exposure to *Dieffenbachia* plant sap can have a full recovery without sequelae after supportive treatment. Eliciting the history from patients regarding the type of plant involved is imperative. Ophthalmologists should be aware of the possibility of crystalline keratopathy developing, even though most patients retain good vision.

## Introduction

*Dieffenbachia* is an ornamental plant widely found in home gardens or commercial plantations. Toxicity caused by contact with the *Dieffenbachia* plant was known as early as the 17th century [1]. The signs of ocular injury include conjunctival chemosis, corneal abrasion, keratoconjunctivitis, and the formation of fine blue crystals in the stroma. Ocular symptoms usually develop in 5-18 hours [2-4]. The main presentations after ocular contact with *Dieffenbachia* plant sap are serious pain, blepharospasm, photophobia, lacrimation, and blurring of vision.

Ocular problems after contact with plant sap of the *Araceae* family, including *Epipremnum aureum, Philodendron, Alocasia, *and *Colocasia* species, have been reported [5]. Here, we describe a patient who presented with crystalline keratopathy after exposure to *Dieffenbachia* plant sap.

## Case presentation

**Figure 1:**
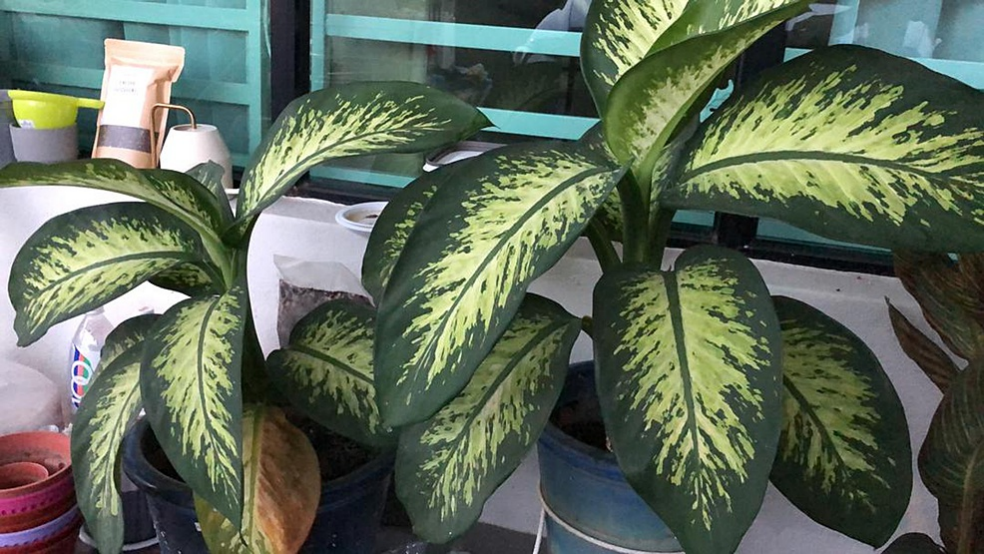
Image of Dieffenbachia plant.

**Figure 2:**
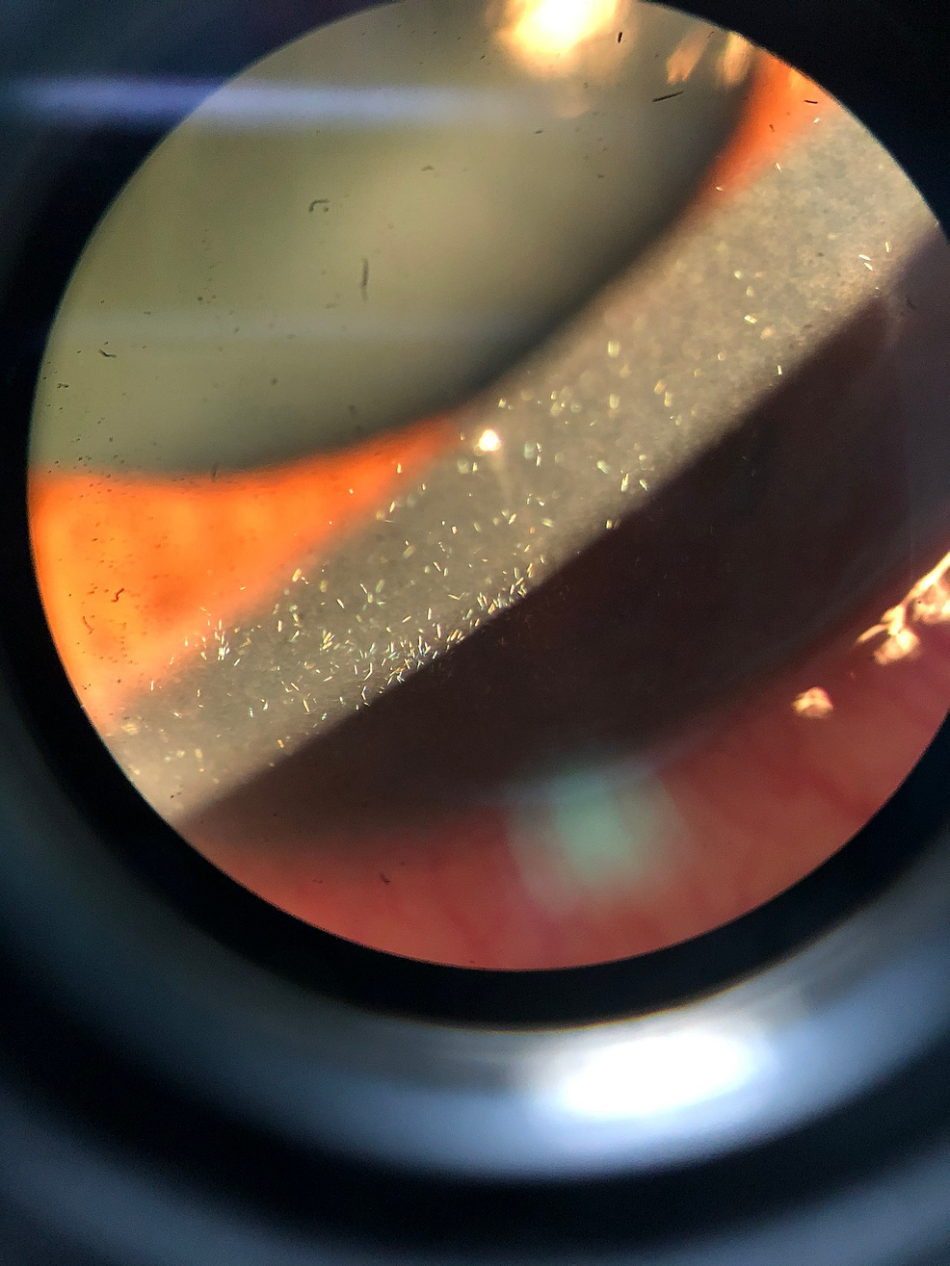
Image of the right cornea on presentation. Tiny needle-like crystals are deposited in the entire epithelial and stromal layer.

A 27-year-old man with underlying diabetes mellitus presented with a one-day history of ocular pain, redness, and tearing. *Dieffenbachia* plant sap had accidentally entered his right eye while he was cutting the grass in his garden one day prior to the presentation. He had immediately irrigated his eye copiously with water, but his symptoms did not improve and he frequently rubbed his eye. Due to persistent ocular pain, redness, and tearing, he decided to seek ophthalmology consultation. The type of plant was identified based on the picture shown by the patient (Figure 1). On presentation, the visual acuity of his right eye was 6/9. Slit-lamp examination revealed fine needle-like oxalate crystals in the epithelial and stromal layer of the cornea (Figure 2). The conjunctiva was moderately injected with epithelial defect over the inferior conjunctiva. The anterior chamber was deep and quiet. The intraocular pressure was 14 mmHg. Posterior segment examination was unremarkable. The fellow eye was completely normal.

**Figure 3:**
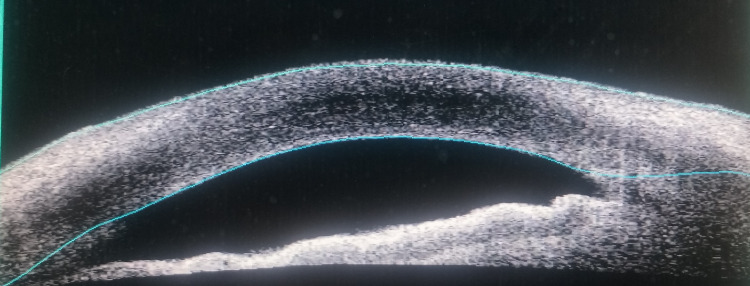
Anterior segment OCT does not visualize hyperreflective structures in corneal layers on presentation. OCT, optical coherence tomography.

Anterior segment optical coherence tomography (OCT) of the right eye did not show hyperreflectivity in the corneal layers at the region containing the oxalate crystals (Figure 3). The patient was treated with 0.1% dexamethasone eye drops and 0.5% levofloxacin eye drops four times daily. The crystals had disappeared from the cornea when he was reviewed one month later. He maintained good visual acuity and no corneal opacity was noted.

## Discussion

*Dieffenbachia* is an ornamental plant found widely in home gardens or plantations. It belongs to the family *Araceae* and grows naturally in tropical regions [2]. The plant has a straight stem and alternate leaves containing white spots and flecks. The active ingredients in the plant sap are calcium oxalate and L-asparaginase [2-4,6]. The calcium oxalate crystals are the component that promotes irritation and inflammation. The severity of ocular inflammation may be related to the plant species, the concentration of sap in contact, and the duration of contact [7]. The literature review showed that children and young adults are commonly affected, probably due to their adventurous behavior and occupation [1,6-10].

Ocular causes of crystalline keratopathy included Schnyder corneal dystrophy, Bietti crystalline dystrophy, infectious crystalline keratopathy, and exposure to *Dieffenbachia* plants. Medical conditions associated with crystalline keratopathy include cystinosis, tyrosinemia, hyperuricemia, multiple myeloma, and monoclonal gammopathy [11]. In contrast, the distinguishing features for Schnyder corneal dystrophy are its progressive course, bilateral involvement, autosomal dominant inheritance, presence of raphides crystals in subepithelium only, and evidence of arcus lipoides [11].

Hsueh et al. reported clinical findings in three cases of ocular injuries caused by the milky latex from *Euphorbia tirucalli* and *Dieffenbachia seguine* [7]. *Euphorbia* sap causes punctate erosion, microbullae, and Descemet’s folds; *Dieffenbachia* sap induces conjunctival chemosis and fine blue crystals in the stroma. Plant sap from the genus *Arisaema* (jack-in-the-pulpit), *Calla palustris* (wild calla), *Colocasia* (elephant ear), *Philodendron*, and *Symplocarpus foetidus* (skunk cabbage) also contain crystals identified as calcium oxalate raphides (needles) and can cause injury similar to that caused by the genus *Dieffenbachia* [7]. The rapier-like shape of the crystals enables easy penetration of the corneal epithelium. The chemical injury is initiated by injury to the tissues and disruption of the epithelial barrier, allowing the penetration of free oxalic acids and plant proteins [1].

A few reports show similarities in the clinical course. Seet et al. reported that the conjunctival injection disappeared by day six after the corneal epithelium had healed [1]. The crystals decreased markedly by day eight, and 75% had dissipated by the end of week two. The remaining crystals slowly disappeared over four to eight weeks [1].

In 1973, Ellis et al. experimented on rabbit corneas by applying a preparation of plant sap on intact cornea [8]. This resulted in severe conjunctivitis, with fluorescein staining of the cornea within 30 minutes. Crystals were seen in the anterior stroma within 24 hours and then involved the posterior cornea at 48 hours. Partial clearing of the crystals was seen on day five, with complete clearing after four to eight weeks. Histological examination of the corneas during the early stages revealed epithelial necrosis, with acute stromal inflammation; however, crystals were not visualized, probably due to the staining process [8].

Chiou et al. reported a similar case that was followed up by serial confocal microscopy at first, fourth, and eighth weeks post-trauma [9]. They reported highly reflective elongated structures in all corneal layers with otherwise undisturbed corneal architecture. There was no inflammatory cell infiltration. Raphide diminution and fragmentation were observed at two months. The authors concluded that confocal microscopy highly improved visualization of the raphides [9]. In our case, the limitation is the unavailability of confocal microscopy to further visualize the raphides in corneal layers.

All cases found in the literature review were treated with supportive measures, such as topical antibiotic eye drops and topical steroids [1,6-10]. One patient was given cycloplegics to relieve the ocular pain [9], while another was treated with oral prednisolone for lid swelling [7]. Complete resolution of crystals in the cornea occurred in one to three months [1,6-10]. The present case highlights the clinical course of crystalline keratopathy induced by *Dieffenbachia* plant sap; therefore, appropriate supportive treatment can be given.

## Conclusions

To conclude, most of the crystalline keratopathies responded well to treatment and the patients had full recovery without sequelae. Ophthalmologists treating patients with ocular contact with plant sap should be aware of the possibility of crystalline keratopathy developing, even though most patients retain good vision. Further in vitro studies on corneas using fractionated components of the plant sap are needed to illustrate the inciting phytochemicals and precise mechanism.
